# The Bidirectional Relationship Between Tuberculosis and Type 2 Diabetes Mellitus: Pathogenesis, Treatment Challenges, and Prevention Strategies

**DOI:** 10.7759/cureus.117316

**Published:** 2026-10-01

**Authors:** Archana Banda, Kim Pedrigal, Savannah Jayne, Jyotsna Chawla

**Affiliations:** 1 Osteopathic Medicine, Dr. Kiran C. Patel College of Osteopathic Medicine, Nova Southeastern University, Clearwater, USA; 2 Foundational Sciences, Dr. Kiran C. Patel College of Osteopathic Medicine, Nova Southeastern University, Clearwater, USA

**Keywords:** bidirectional relationship, diabetes and tuberculosis co-infection, immune dysfunction, inflammation, prevention, treatment regimen, tuberculosis, type 2 diabetes mellitus (dm)

## Abstract

Pulmonary tuberculosis (TB) and type 2 diabetes mellitus (T2DM) are significant global health burdens that independently impair immune function. When present concurrently, they exacerbate disease severity, reduce treatment effectiveness, and increase mortality. Recent literature suggests a growing bidirectional relationship between these conditions. The purpose of this investigation is to evaluate how T2DM-induced immune dysregulation increases susceptibility to TB and worsens clinical outcomes. A literature review was conducted using PubMed, focusing on articles related to cytokine signaling, immune modulation, T2DM, and tuberculosis. T2DM contributes to impaired immunity primarily through hyperglycemia-induced suppression of cytokines, such as tumor necrosis factor-alpha (TNF-α) and various interleukins (IL-1β, IL-6, and IL-12). This dysregulation compromises macrophage and T-cell responses, increasing the risk of TB activation and reducing treatment efficacy. The rising incidence of both diseases in the United States highlights the urgent need to understand this mechanistic overlap to improve therapeutic strategies. T2DM serves as an independent risk factor for TB due to its immunosuppressive metabolic environment. Recognizing these mechanisms can inform clinical decisions and improve the management of co-affected patients.

## Introduction and background

Pulmonary tuberculosis (TB), caused by *Mycobacterium tuberculosis*, has been feared as one of the deadliest infectious diseases known. In 2023, 1.25 million people globally died from this infection, making it the world’s leading cause of death from an individual pathogen [1]. The signs and symptoms of TB include prolonged cough with blood, fever, night sweats, and weight loss [1]. Despite TB being a significant global health challenge, it is preventable and curable with a strict drug regimen lasting six months, specifically two months of HRZE/four months of HR regimen [2]. However, this bacterium has recently mutated to an additional form: multidrug-resistant TB (MDR-TB), further complicating the treatment process, which necessitates expert guidance on treatment choices [2]. This form is resistant to at least isoniazid and rifampin, the two most effective first-line TB drugs, while extensively drug-resistant TB (XDR-TB) has additional resistance to any fluoroquinolone and a second-line injectable (amikacin, capreomycin, and kanamycin) [2].

TB poses a significant burden by itself, making co-infections with other diseases such as human immunodeficiency virus (HIV), hepatitis B, and type 2 diabetes mellitus (T2DM) lead to suboptimal outcomes [3-5]. These co-infections can be explained by overlapping risk factors. TB and many of its co-infections occur in lower/middle-income and resource-poor countries such as India, Pakistan, and the Democratic Republic of the Congo [6]. The incidence of T2DM is also increasing in lower-income regions, and the age-standardized disability-adjusted life year (DALY) rates are highest in regions such as Latin America, sub-Saharan Africa, and Southeast Asia [7]. As such, the probability of the incidence rate of comorbid TB and T2DM is increasing in sub-Saharan Africa and South Asia. 

While the relationship between HIV and TB has been widely studied, the relationship between T2DM and TB is an emerging area of study. DM was the most reported medical risk factor among people with active TB disease, which is further complicated when patients with diabetes and TB are nine times more likely to have treatment failure than patients with solely TB [4,5]. Based on current research, we hypothesize that patients with diabetes are more likely to develop active TB and experience worse clinical outcomes compared with those without diabetes. This review aims to elucidate the bidirectional relationship between TB and T2DM, focusing on immune dysregulation mechanisms, treatment challenges, and prevention strategies.

## Review

Methods

This narrative review aimed to gain a clearer understanding of the T2DM and TB syndemic, with an emphasis on molecular basis of disease mechanisms, bidirectional and shared mechanisms, treatment regimens for TB and T2DM, and treatment and management challenges for co-infection. This was conducted using PubMed and Google Scholar with keywords including “Type 2 Diabetes Mellitus,” “Tuberculosis,” “cytokine signaling,” and “immune modulation.” Relevant articles from the past 15 years were included. However, two articles included in this review were published earlier; they were retained because they contained critical information on tumor necrosis factor-alpha (TNF-α) and pyridoxine that remained relevant to the scope of this review.

Molecular basis of disease mechanisms

Tuberculosis 

Pulmonary TB begins when a person inhales *M. tuberculosis* bacteria from an infected person through airborne droplets released by coughing, talking, or breathing [8]. The bacteria travel through the airways, infecting cells along the way, and eventually reach the alveoli, where they infect alveolar macrophages [9]. These infected macrophages can then move to the lung tissue or lymph nodes, triggering an immune response. The body responds by forming a granuloma, a cluster of infected and uninfected immune cells, including macrophages, monocytes, neutrophils, and T cells [8]. In most cases, the infection is contained at this stage, and the person remains asymptomatic. However, if the immune system fails to control it, the infection can progress to active primary or reactivation TB [8]. In such cases, the granuloma can break down into a caseating granuloma, which supports bacterial growth and allows *M. tuberculosis* to spread through the lymphatic system or airways [9]. The bacteria can evade the immune system for years due to various virulence factors, such as mycolic acid, trehalose-based molecules, Toll-like receptor 2 (TLR-2) modulators, and cytokine inhibitors [9]. These mechanisms contribute to the ongoing challenge of developing an effective TB vaccine.

Type 2 Diabetes Mellitus 

T2DM occurs when the beta-islet pancreatic cells can no longer produce sufficient insulin to support the body’s needs, and the body’s tissues become insensitive to the insulin the pancreas produces. Defective beta cells can occur before the development of obesity and insulin resistance. Furthermore, 1,000 genetic defects associated with T2DM may indicate a genetic component of T2DM [10]. Additionally, new research is shedding light on the role of microRNAs (miRNAs) in the development of T2DM; these non-coding molecules control the regulatory process of metabolic events. Increased levels of certain miRNAs (miR-27a and miR-320a) may lead to increased fasting blood glucose levels, which promote the development of T2DM [11]. Also, the conversion of proinsulin to insulin is decreased in T2DM, further complicating the body’s ability to produce active insulin [10]. Insulin is allowed to enter the cell through the GLUT4 transporter. Recent literature supports that dysregulation of this pathway leads to insulin resistance and remains a major contributor to the development of T2DM [10]. Another factor that negatively impacts glucose homeostasis is gut dysbiosis, which compromises the intestinal barrier and facilitates systemic inflammation. To counteract this, probiotics can be used to alleviate critical mechanisms such as insulin resistance and dysfunction of glucose metabolism in T2DM pathogenesis and complications [12]. 

Also, T2DM is closely linked to obesity and a sedentary lifestyle. Although it is mainly seen in adults, in recent years, there has been a rise in T2DM during childhood, especially among the non-Hispanic Black population [12]. According to the American Diabetes Association, the diagnostic criteria for diabetes in a non-pregnant individual require an A1C greater than or equal to 6.5% or fasting plasma glucose greater than or equal to 126 mg/dL or a random plasma glucose greater than 200 mg/dL in an individual with classic symptoms of hyperglycemia [13].

Bidirectional and shared mechanisms

Chronic Inflammation and Cytokine Dysregulation in T2DM

T2DM causes multiple issues in the immune regulation pathways, ultimately leading to lower rates of immunity in T2DM patients, which in turn serves as a risk factor for developing pulmonary TB. T2DM is associated with a state of chronic inflammation in the body due to the recruitment, accumulation, and activation of pro-inflammatory macrophages in metabolic tissues [14]. Macrophages and many other types of cells involved in the immune system secrete proteins known as cytokines, which have numerous effects on different cells and potentiate the immune response. This state of chronic inflammation further exacerbates the effects of hyperglycemia and insulin deficiency because of increased TNF-α, which reduces insulin sensitivity [14]. TNF-α, a key cytokine from macrophages and natural killer cells, drives acute and chronic inflammation and may affect the central nervous system. This cytokine leads to a vicious cycle of inflammation and hyperglycemia, further worsening the immune dysregulation in T2DM.

Cytokine Dysregulation in TB

TB infection triggers the body’s immune system, leading to increased levels of circulating cytokines such as TNF-α. TNF-α can lead to a temporary suppression of the insulin receptor kinase, resulting in insulin resistance and hyperglycemia [15]. Therefore, if a person with T2DM becomes infected with active TB, it can lead to worsening of their glucose levels and potentially increase their insulin requirements. Additionally, this transient hyperglycemia could have the ability to distinguish those at risk for T2DM in the future [16]. A study conducted in the UK found a two to five times increase in the rate of T2DM occurring after a TB infection [16]. This demonstrates the fact that there is a bidirectional relationship between TB and T2DM not only through the hyperglycemia effect mentioned above but also possibly through the effects of weight gain during treatment. Persons with active TB generally lose weight during the infection, and during treatment (especially with hyperglycemic medication) may gain it back; this increase in body fat may lead to the development of T2DM [16].

Another possible pathway that leads to insulin resistance among TB-infected individuals is the effect of hypertrophic adipose tissue on the dysregulated production of cytokines like TNF-α and monocyte chemoattractant protein-1 (MCP-1) [17]. MCP-1 is a pro-inflammatory cytokine released by adipose tissue. In TB, adipose tissue is found to have increased production of MCP-1, leading to insulin resistance and consequently T2DM [17].

Interleukins and Their Role in TB Susceptibility 

Another way T2DM dysregulates the immune system is by decreasing the production of interleukins (IL), which are cytokines that function to modulate growth, differentiation, and activation during inflammatory and immune responses. High blood glucose levels lead to lower IL-1β, IL-2, IL-6, IL-10, and IL-17A levels, which negatively impact immune function [18]. Peripheral blood mononuclear cells (PBMCs) in T2DM patients had significantly more intracellular bacteria than their healthy counterparts [19]. Notably, IL-12 production was impaired specifically during a TB infection [19]. Another study demonstrated that reactive oxygen species (ROS) production may be inhibited by increased levels of a protein known as resistin in severe cases of TB and T2DM [20]. ROS serve a critical role in immunity by killing intracellular bacteria. Consequently, high levels of them are crucial during an infection of intracellular bacteria, such as TB. These various mechanisms by which T2DM weakens the immune system by decreasing the number of cytokines and ROS in affected patients illustrate how it serves as an independent risk factor for developing pulmonary TB (Figure 1).

**Figure 1:**
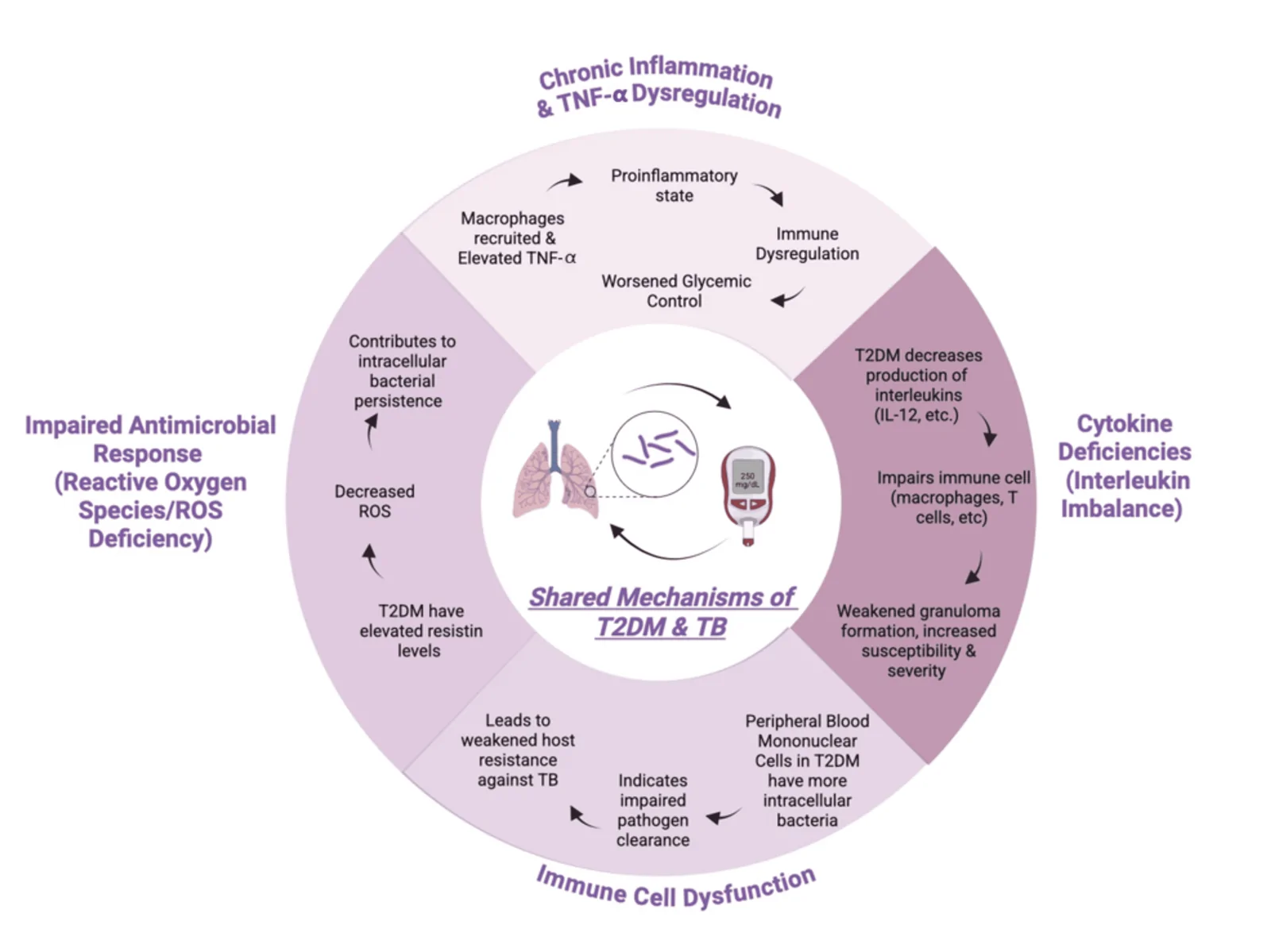
The four key shared mechanisms between T2DM and TB are chronic inflammation, cytokine deficiencies, immune cell dysfunction, and impaired antimicrobial response. These synergistic effects increase the likelihood of a patient with T2DM developing active pulmonary TB. This figure was created with BioRender.com. T2DM: type 2 diabetes mellitus; TB: tuberculosis; TNF-α: tumor necrosis factor-alpha; ROS: reactive oxygen species; IL: interleukin.

Treatment regimen for TB

The current primary agents used to treat TB are rifapentine, isoniazid, pyrazinamide, and ethambutol, all of which have significant adverse effects shown in Table 1. According to the Centers for Disease Control and Prevention, there are various treatment regimens for TB [2]. These treatment regimens vary depending on the drug or combination of drugs used and the duration of the therapy. Examples of these treatment regimens include the four-month rifapentine-moxifloxacin TB treatment or the six- or nine-month RIPE (rifampin, isoniazid, pyrazinamide, and ethambutol) TB treatment [2]. Each of the FDA-approved first-line anti-TB agents has varying mechanisms of action [21]. Rifampin reversibly inhibits DNA-dependent RNA polymerase. Isoniazid is a prodrug that inhibits the biosynthesis of mycolic acid. Pyrazinamide inhibits trans-translation and coenzyme synthesis. Ethambutol inhibits arabinosyl transferases and prevents the biosynthesis of the cell wall [21]. The shared adverse effect that must be monitored is hepatotoxicity for all RIPE drugs. The strategy for these plans includes a Directly Observed Therapy Short-course (DOTS) where a healthcare worker directly observes the patient taking their medication to improve adherence to the treatment plan [22]. Treatment plans can also differ depending on whether the TB is latent, active, or multidrug-resistant, as shown in Table 1. Additionally, accommodations may be made if the patient is HIV-positive, pregnant, or a child [2].

**Table 1:** Summary of TB treatment regimens and dosages. TB: tuberculosis; RIF: rifampin; INH: isoniazid; P: rifapentine; PZA: pyrazinamide, EMB: ethambutol; B: bedaquiline; Pa: pretomanid; L: linezolid; M: moxifloxacin.

Type of TB	Treatment regimen	Drugs	Time span	Total doses	Dosage	References
Latent TB	9H	Isoniazid (H)	Nine months, daily	270	Adults: 5 mg/kg Children: 10–20 mg/kg Maximum dose: 300 mg	[23]
	3HP	Isoniazid (H) Rifapentine (P)	Three months, once weekly	12	Isoniazid, adults and children ≥12 years old: 15 mg/kg rounded up to the nearest 50 or 100 mg (900 mg maximum) Rifapentine is dependent on weight	
	4R	Rifampin (R)	Four months, daily rifampin	120	Adults: 10 mg/kg	
	6H	Isoniazid (H)	Six months, daily	180	Adults: 5 mg/kg (daily)	
			Six months, twice weekly	52	Adults: 15 mg/kg (twice weekly)	
Active TB	RIPE	Rifampin (RIF) Isoniazid (INH) Pyrazinamide (PZA) Ethambutol (EMB)	Initiation phase: Two months (8 weeks oral, daily) of rifampin, isoniazid, pyrazinamide, and ethambutol Continuation phase: Four months (18 weeks oral, daily) of isoniazid and rifampin	Initiation phase: 56 Continuation phase: 126	RIF: 10 mg/kg (600 mg max) INH: 5 mg/kg (300 mg max) PZA: 15-30 mg/kg (2 g max) EMB: 15-25 mg/kg (1.6 g max)	[21,24]
MDR TB	BPaLM	Bedaquiline (B), pretomanid (Pa), linezolid (L), moxifloxacin (M)	Six months	632	B: 400 mg daily for two weeks, then 200 mg three times/week for subsequent 24 weeks Pa: 200 mg daily for 26 weeks L: 600 mg daily for 26 weeks M: 400 mg daily for 26 weeks	[25,26]

Treatment regimen for T2DM

Treating a patient with T2DM also requires a multidisciplinary approach, including lifestyle changes in diet and physical activity, as well as medication to control glycemic levels. These medications can include metformin, sulfonylureas, and dipeptidyl peptidase-4 (DPP-4) inhibitors [27]. Table 2 lists some of the antidiabetic drugs with their mechanism of action and potential adverse effects. Metformin, a first-line medication for the treatment of T2DM, is shown in Table 2. This agent bypasses hepatic metabolism and is safe to use for patients with pre-existing T2DM and TB [28]. In a study done with Taiwanese patients with TB (with and without pre-existing T2DM), researchers found that those taking metformin showed decreased mortality compared to non-metformin users. Therefore, there is potential use of metformin as an adjunctive therapy for TB treatment in patients with pre-existing T2DM [29]. Despite the clear guidelines for TB and T2DM treatment options, the bidirectional relationship between TB and T2DM can complicate the treatment of patients with pre-existing T2DM or TB. Those with T2DM have triple the risk of acquiring active TB. Diabetic patients with active TB are also two to three times more likely to have complications during the treatment [30,31]. Currently, there is no difference in the treatment plans for TB in patients with and without T2DM [32]. Generally, having optimal glycemic control concurrently with TB treatment is essential for improving treatment outcomes [33]. Also, drug-drug interactions between antidiabetic drugs and anti-TB drugs during concurrent treatment must be monitored.

**Table 2:** Overview of antidiabetic drugs. AMPK: adenosine monophosphate-activated protein kinase; DPP-4: dipeptidyl-peptidase 4, SGLT-2: sodium-glucose co-transporter 2.

Medication (Drug class)	Dosage	Mechanism of action	Adverse effects	References
Metformin (biguanides)	500 to 1,000 mg tablets twice a day, oral	Reduces hepatic gluconeogenesis by increasing AMPK activity	Gastrointestinal issues (e.g., diarrhea) (12% to 53%), nausea and vomiting (7% to 26%)	[34,35]
Glipizide (sulfonylureas)	2.5 mg to 10 mg tablet, single dose or two divided doses	Stimulates insulin secretion by binding to K-ATP channels of the pancreas	Syncope (<3%), dizziness (2% to 7%), nervousness (4%)	[34,35]
Sitagliptin (DPP-4 inhibitors)	25 mg, 50 mg, or 100 mg once daily	Inhibits enzyme DPP-4	Varies depending on the medication; sitagliptin: nasopharyngitis (5%), hypoglycemia (1%)	[34,35]
Canagliflozin (SGLT-2 inhibitors)	Initially, 100 mg daily, gradually increased to 300 mg daily	Inhibits SGLT-2	Fungal vaginosis (7% to 8%), urinary tract infection (6%)	[34,35]

Treatment challenges for co-infection

Pre-existing T2DM may interfere with TB treatment by affecting gut motility and metabolism, leading to poor drug absorption and a weaker therapeutic response [28]. Liver function in patients with diabetes is an area of concern, especially in the case of a specific anti-TB agent, rifampin. Rifampin induces cytochrome P450 enzymes and glycoproteins that metabolize anti-T2DM medications, reducing their effectiveness in maintaining glycemic control. Consequently, T2DM treatment is complicated, and the possibility of hepatotoxicity is exacerbated (Table 2) [28]. Worsening diabetic neuropathy could be another complication that may result from anti-TB drugs, notably isoniazid (INH), which itself induces the adverse effect of peripheral neuropathy [22]. Also, patients with pre-existing T2DM may have a higher starting bacterial load or slower response to treatment, which could result in an increased chance of acquiring MDR TB [22,32]. 

While some studies report altered pharmacokinetics and pharmacodynamics with the antibiotics used for TB treatment and T2DM medications, at least one study did not show statistically significant differences in plasma concentrations between patients with T2DM and TB versus TB-only patients when treated with rifampicin [36]. These conflicting reports show an emerging need for additional studies exploring treatment plans for patients with concurrent TB and T2DM. 

In addition to pharmacological treatments, adjunctive therapies may support the management of TB and T2DM. Metformin, a first-line T2DM medication, has shown promise in reducing TB-related mortality in patients with TB-T2DM comorbidity, potentially serving as an adjunctive therapy due to its favorable hepatic metabolism profile [29].

Management strategies for co-infections

Given the treatment challenges that patients with T2DM and TB face, it is critical for these patients to be monitored closely to prevent further clinically significant ramifications. Routine liver function tests are crucial since all anti-TB agents share the adverse effect of hepatotoxicity. Also, there are unique adverse effects for each drug in the RIPE therapy that clinicians and patients should be aware of. For instance, rifampin is a CYP inducer, which impacts the metabolism of other drugs. Consequently, clinicians should alert patients taking rifampin that medications also metabolized by the CYP450 system will have reduced clinical efficacy [28]. Notably, rifampin significantly induces CYP3A4 and P-glycoprotein, resulting in reduced circulating levels of oral hypoglycemics such as glipizide or linagliptin. This causes suboptimal glycemic control. Unlike agents metabolized by the CYP enzymes, metformin remains the preferred foundational agent since it is cleared by the kidneys and not subject to CYP-mediated drug interactions with rifampin.

As previously stated, isoniazid can also cause severe peripheral neuropathy since it competitively inhibits pyridoxine's actions [37]. However, this irreversible side effect can be avoided with mandatory supplementation of vitamin B6 (pyridoxine) 25-50 mg daily. In high-risk populations, such as individuals with diabetes, mitigating peripheral neuropathy, especially given their increased likelihood of existing vascular complications, is paramount to patient safety. Pyrazinamide can also cause arthralgia, hyperuricemia, and is a potential teratogen [21]. It is imperative to monitor uric acid levels, as existing literature generally concurs that hyperuricemia and gout are associated with a heightened risk of developing diabetes [38]. Consequently, in patients taking pyrazinamide treatment for TB, clinicians must monitor and manage plasma glucose and uric acid levels to mitigate the risk of developing diabetes.

Another well-established side effect of the RIPE therapy is ethambutol-induced optic neuropathy. This devastating complication has been shown to occur in 1% of patients taking this agent, according to the World Health Organization [39]. Clinicians should alert patients that an increased ethambutol dose increases the risk of this rare complication. Patients must immediately discontinue if they develop any signs of optic neuropathy. As explored above, having diabetes as a comorbidity complicates TB therapy. Therefore, clinicians must screen TB patients for diabetes, especially if they have risk factors, to optimize patient prognosis [40].

In 2011, the World Health Organization and International Union Against Tuberculosis and Lung Disease published the Collaborative Framework for Care and Control of Tuberculosis and Diabetes [41]. The management strategies recommended early detection and screening tests, as well as integrative coordination in the care for TB and T2DM. Screening patients with known diabetes for TB when they complain of symptoms such as chronic cough and other TB symptoms, as well as screening all TB patients for diabetes at the beginning of their treatment, should be implemented.

As clinicians managing co-infection of TB and T2DM, it is important to evaluate the patient for the aforementioned side effects of the various anti-TB and anti-T2DM medications being concurrently taken. Strict glycemic control should be maintained in these patients with an HbA1c target of <8%, although a target of about 7% is generally recommended [42]. Collaborative care is also emphasized to maintain anti-TB and anti-T2DM drug adherence. Ensuring patients have an understanding of the treatment regimens for both diseases, the chronic nature of T2DM, and continuing care even after TB treatment has ended [42].

Discussion

Exploring the bidirectional relationship between T2DM and TB has elucidated a cycle wherein one disease state worsens the risk of developing the other disease while also increasing challenges in treatments if co-infection was present. T2DM's chronic state of inflammation leading to impaired adaptive and innate immune functions creates a more susceptible patient for TB to attack. The treatment regimens that are well established to treat TB unfortunately have adverse effects when paired with antidiabetic drugs used to treat T2DM. As both diseases are well studied and their treatment regimens established, future explorations into the topic could discuss the role of bidirectional screening when diagnosed with one disease. Clinicians could also consider having a lower threshold of screening when symptoms arise if a patient has a known history of either disease. Notably, clinicians should be careful about diagnosing T2DM using HbA1c or transient fasting glucose when patients are presenting acutely with TB. In these clinical situations, it is critical for these patients to be re-assessed after an acute phase of TB.

Limitations

The limitations of this review should be considered when interpreting the findings. First, the literature search relied on a predefined set of keywords, including "cytokine signaling," which may have excluded studies analyzing relevant bidirectional mechanisms using different terminology. To be included in the narrative review, the study had to be from the past 15 years, which may have limited the search criteria. However, two references outside this time span were included: TNF-α and pyridoxine supplementation during isoniazid therapy. Consequently, some contributing pathways or emerging evidence may not have been captured. Second, due to the evolving research centered on this syndemic, additional mechanisms and therapeutic targets may have been identified after the completion of the literature search. Third, only English-language publications were included, introducing the potential for language bias and limiting the inclusion of relevant studies published in other languages. Finally, this review did not include a formal statistical meta-analysis or quantitative assessment of study quality and risk of bias.

## Conclusions

The bidirectional relationship between TB and T2DM, driven by immune dysregulation and poor treatment outcomes, necessitates integrated treatment and prevention strategies. The shared mechanisms between the two conditions, such as cytokine deficiencies, impaired antimicrobial response, chronic inflammation, and immune cell dysfunction, indicate an increased likelihood that patients with type 2 diabetes will develop active pulmonary TB. Further research is needed to optimize treatment regimens, such as randomized controlled trials to assess metformin's role as an adjunctive therapy for TB in patients with T2DM. In regions with a high disease burden, policymakers should prioritize and implement bidirectional screening.
